# A case of conservatively managed idiopathic spinal cord herniation presenting with low-pressure headache

**DOI:** 10.1093/jscr/rjae063

**Published:** 2024-03-08

**Authors:** Marco Mancuso-Marcello, Joseph Frantzias, Carl Hardwidge

**Affiliations:** Department of Radiology, The Royal London Hospital, Barts Health NHS Trust; Department of Neurosurgery, Stanford University Medical Centre; Department of Neurosurgery, The Royal Sussex County Hospital, University Hospitals Sussex NHS Trust

## Abstract

Idiopathic spinal cord herniation presenting with low-pressure headache is extremely rare. We present a case of thoracic ventral spinal cord herniation in a 35-year-old lady who presented with low-pressure headaches. To our knowledge, this is only the fourth case described in the literature of spontaneous ventral cord herniation presenting in this way. The patient was managed conservatively with no manifestation of focal neurological symptoms at 12-month follow-up. The proposed aetiology of spontaneous ventral cord herniation is an initial CSF leak via a dural defect, through which the cord subsequently also enters blocking the CSF leak. We endorse a conservative approach for patients who present similarly, secondary to the above pathophysiology.

## Introduction

Spinal cord herniation (SCH) is a rare condition in which the spinal cord herniates through a defect in the dura, presenting most often with Brown-Sequard syndrome. While it occurs almost exclusively on the ventral aspect of the thoracic spine between Levels T3–T7, the exact pathogenesis is the source of active speculation and the vast majority of cases are idiopathic and are treated with surgical repair of the dural defect. Here, we present the case of a 35-year-old lady who presented unusually with sudden-onset low-pressure headache, had normal cord signal on magnetic resonance imaging (MRI) with no focal neurological symptoms, and was managed conservatively.

## Case report

A 35-year-old right-handed lady developed a sudden-onset headache while lifting a piece of luggage. She described it as 10/10 in severity, and it was associated with nausea and vomiting. Subsequently, the headache was relieved by lying down flat, but it would reoccur if the patient lifted the head or stood up. The severity of the symptoms was such that the patient had to remain lying flat while eating. Following personal research, the patient started taking caffeine that partially ameliorated the headache.

Four days after the index onset of headache, the patient presented to the emergency department. Initial blood tests and a CT scan were unremarkable. Lumbar puncture was technically difficult and could only be performed in a sitting position. This yielded pauce CSF outflow with unmeasurable opening pressure.

Two weeks prior to the index onset of headache, the patient reported having fallen onto her lower back, but the subsequent pain resolved over the subsequent days with no sequelae. The patient’s past medical history is notable for spinal anaesthesia 7 years prior during delivery of her first child after which she was admitted hospital with bad headaches which lasted for 2–3 weeks. The patient reports no other significant headache history such as migraine.

**Figure 1:**
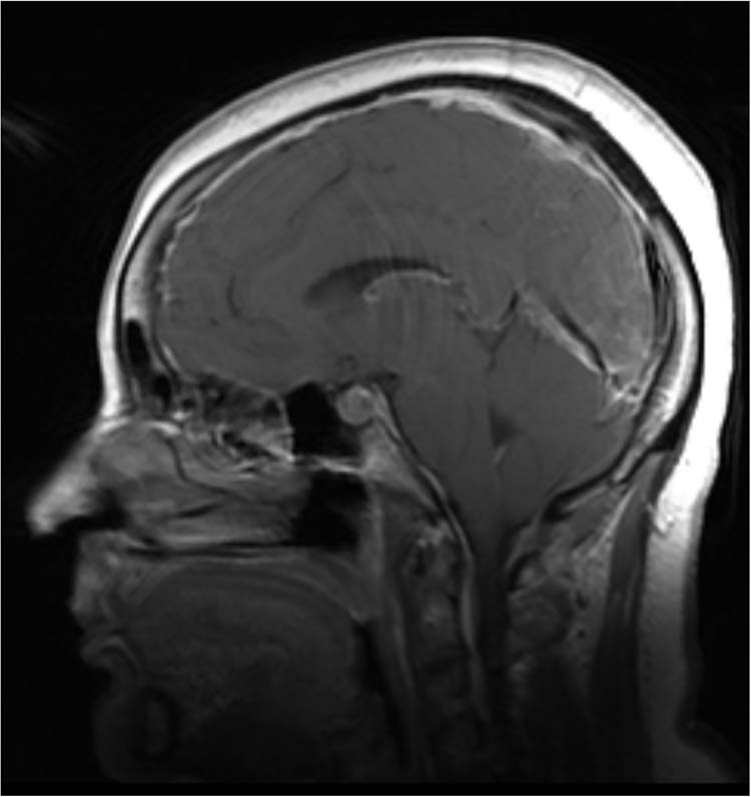
Illustrative sagittal gadolinium enhanced T1 MRI of the brain demonstrating the sequalae of intracranial hypotension, the likes of which may be precipitated by a ventral spinal dural defect; there is smooth pachymeningeal enhancement, partial effacement of the cortical sulci, sagging cerebellar tonsils, a full appearing pituitary gland and slight prominence of the straight sinus; illustrative sagittal gadolinium enhanced T1 MRI of the brain, demonstrating the sequalae of intracranial hypotension, the likes of which may be precipitated by a ventral spinal dural defect; case courtesy of Behrang Amini, from the case rID: 36019 (https://radiopaedia.org/cases/36019?lang=gb)

**Figure 2:**
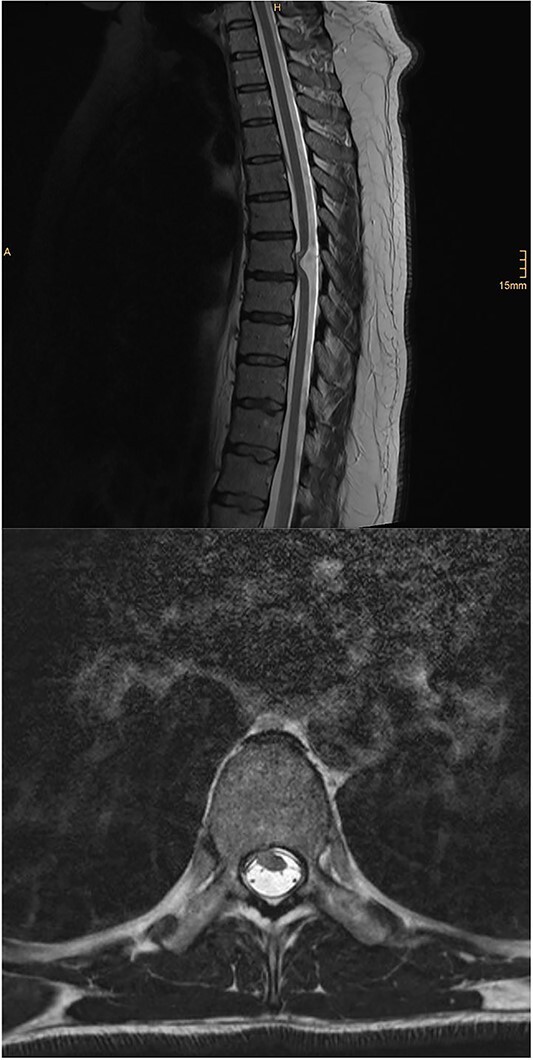
Illustrative sagittal and axial T2 MRI of the spine demonstrating ventral SCH; there is focal anterior displacement of the spinal cord out of the dural sac through a dural defect at the T7 level; unlike in the present case, these images demonstrate an ill-defined focus of T2 hyperintensity within the cord at the T7 level; case courtesy of Kamran Hajiyev, from the case rID: 76419 (https://radiopaedia.org/cases/76419?lang=gb)

MRI of the brain (see Fig. 1) revealed features of low intracranial pressure. An MRI of the spine (see Fig. 2) revealed focal thoracic herniation of the ventral cord through the thecal sac with CSF signal in the anterior epidural space. No cord signal abnormality was demonstrated. Neurological examination was unremarkable with no numbness or weakness of the lower limbs.

The patient was discharged from hospital. At 6- and 12-month reviews, she reported improvement of the headaches to 4/10 severity, especially on taking caffeine. Neurological examination, including that of the lower limbs, remains unremarkable. She continues to attend follow-up at yearly intervals but she does not currently desire any form of surgical intervention.

## Discussion

SCH is a rare condition in which the spinal cord herniates through a defect in the dura. In this report, we present a case of idiopathic ventral SCH which is unusual in at least 2 ways. The first is that the patient presented with symptoms of low intracranial pressure headache, but none of the traditional symptoms of Brown Sequard syndrome or paraparesis. This is seen in only three other cases in the literature. The first describes a 21-year-old man who had a 3-year history of intermittent low-pressure headaches consistent with intracranial hypotension. Eventually, the headaches resolved, but he developed myelopathy due to a SCH. In this case, the authors hypothesize that the progressive SCH through a spontaneous dural tear sealed the site of cerebrospinal fluid leak, causing the resolution of headaches [1]. The second case describes a 53-year-old woman who initially presented 15 years prior with an episode of intense postural headache. Six months later, she developed sensory changes in her right leg and subsequent myelopathy that progressed in severity for several years [2]. The third case describes a 36-year-old man who presented with headache, pain in the left side of the back and chest, and left sided foot drop. He had a 3-year history of frequent, severe throbbing headaches starting at the nape of the neck and radiating all over the head, with occasional vomiting [3]. The present case differs from all three in that the low-pressure headache occurred as an isolated presenting symptom. However, we would argue that the pathophysiology of our case follows a similar course to that suggested by the authors of the first case above. We hypothesize that the low-pressure headache was precipitated by a CSF leak which began at an indeterminate time point. This CSF leak would have developed through a dural defect that could either be congenital or acquired, perhaps traumatically. The gradual resolution of the headaches could be explained by the plugging of the dural defect by the cord, as is hypothesized in the first case above; this is seen on MRI as cord herniation. The intact signal intensity of the cord reconciles the lack of focal neurological deficits in the current patient.

The second way in which the present case is unusual is that it was conservatively managed. Summers *et al*.’s [4] clinical review of 174 patients with idiopathic SCH found that, of the 15 cases which were conservatively managed, 100% had no improvement or worsening of neurological compromise over time (mean follow-up = 33 months). However, this stability of symptoms in the conservatively managed is a potentially misleading statistic, given that progression of neurological symptoms itself would be a strong prompt to escalate to surgical management. Of the surgically managed patients, there was an improved neurological outcome in 74%, unchanged result in 18%, and worse outcome in 8%. With this in mind, we would recommend surgical intervention to be absolutely indicated with the onset of focal neurological compromise, but patients presenting with just postural headache and a normal cord may be good candidates for an initial watch-and-wait approach.
